# A Manual of Auscultation and Percussion

**Published:** 1849-04

**Authors:** 


*A Manual of Auscultation and Percussion,* by M. Barth, Agrégé to the Faculty of Paris, &c. &c. and M. Henry Roger, Physician to the Bureau Central of the Parisian Hospitals, &c. Translated with additions, by Francis E. Smith, M. D. Second edition. Philadelphia: Lindsay &Blakiston, 1849.

The great value of auscultation and percussion is universally admitted, and nothing is more necessary to the student, and even physician, than a condensed summary of the facts already ascertained as to the character and meaning of the many sounds distinguishable in the working of the complex human machinery, and the best manner of perceiving them. For this purpose, the little work before us is designed, and for this purpose it is admirably adapted. To the students of auscultation and percussion, it is invaluable—and we recommend it to all who desire exact knowledge of the subject.

